# Can viewing a 3D movie improve visual function in children with a history of amblyopia and neurotypical children?: A pilot study

**DOI:** 10.1371/journal.pone.0305401

**Published:** 2024-06-25

**Authors:** Laura Asensio-Jurado, Marc Argilés, Lluïsa Quevedo-Junyent, Clara Mestre, Dennis M. Levi

**Affiliations:** 1 Centre for Sensors, Instruments and Systems Development (CD6), Universitat Politècnica de Catalunya, Terrassa, Spain; 2 Departament d’Òptica i Optometria, Universitat Politècnica de Catalunya, BarcelonaTech (UPC), Terrassa, Spain; 3 Hospital Universitari Mutua Terrassa, Terrassa, Spain; 4 Herbert Wertheim School of Optometry & Vision Science, University of California, Berkeley, Berkeley, CA, United States of America; The Ohio State University, UNITED STATES

## Abstract

**Purpose:**

The aim of this pilot study was to determine whether viewing an immersive 3D movie with large disparities in a cinema resulted in improved visual acuity (VA), stereoscopic depth perception (ST), and improved eye alignment in residual amblyopic children and children without amblyopia.

**Methods:**

A total of 24 children aged between 5 and 12 years with a history of anisometropic and/or strabismic amblyopia, that had been previously treated and who currently have residual amblyopia (N = 14), and in children with typical development without amblyopia (N = 10) viewed the movie in 3D Sing 2 in a cinema for 110 minutes. Visual acuity, stereoacuity and ocular deviation were assessed before viewing the movie, and three months later. Stereoacuity and ocular deviation were also measured immediately after viewing the movie.

**Results:**

We observed an improvement in visual acuity in the non-dominant (amblyopic) eye 3 months after viewing the movie in the amblyopic group (P<0.001). Stereopsis improved immediately after viewing the movie (P = 0.02), and after 3 months by ≈ 40% (P = 0.01). Moreover, improvements in stereopsis were also observed in children without amblyopia (P = 0.04). No significant changes in ocular deviation were observed in either group.

**Conclusions:**

These pilot results suggest that brief exposure to large disparities by viewing a 3D movie in a cinema can help to improve stereopsis and visual acuity in children aged 5‒12 years with previously treated amblyopia, and provide a rationale for a randomized clinical trial.

## Introduction

The presence of strabismus (a turned eye) and/or amblyopia (lazy eye) in childhood is the most common cause of impaired binocular vision, and consequently, reduced or absent stereoacuity. Conventional clinical treatment for children with amblyopia consists of correcting any refractive error (i.e., optical correction) followed by penalizing or patching the dominant (non-amblyopic) eye (occlusion). Both optical and occlusion treatments have proven to be effective in improving visual acuity in all types of amblyopia [1–7]. In addition, slight improvements in stereoacuity have also sometimes been observed after correcting the refractive error [8, 9]. However, conventional treatment does not always provide complete recovery of visual acuity and stereoacuity even after long periods of treatment [5, 6, 10–15]. Additionally, even after the visual acuity deficit has been resolved, stereo depth perception is frequently impaired [16]. Under ordinary everyday conditions, with both eyes open, impaired stereo depth perception is the most common visual problem exhibited by people with amblyopia. Impaired stereopsis may result in deficits in visually guided hand movements, fixation instability, poor vergence control and alter gaze strategies for navigation (see Niechwiej-Szwedo et al., 2019; Levi, 2020 and 2022 for recent reviews) [17–19]. For this reason, new approaches have been developed that seek to restore binocular vision and focus on the rehabilitation of stereopsis in individuals with amblyopia and/or strabismus with encouraging results. These new approaches include perceptual learning (PL) [20–24], action video games [13, 24, 25] and 3D movie viewing [26].

The current study was inspired by the personal experience of the late Bruce Bridgeman, a vision scientist with deficient stereo depth perception all his life, who improved it after watching the 3D movie *"Hugo”* in a real cinema [27]. Bridgeman speculated that his improvement was the result of “sustained attention to varying high-disparity stereoscopic challenges in an engaging immersive environment” [17, 27]. Subsequent studies suggested that playing 3D videogames with large binocular disparities can help to restore stereo vision in both neurotypical adults [28] and in adults with amblyopia [26]. In light of this background, the objective of this study was to determine whether viewing a 3D movie with large disparities in a cinema could be effective to improve stereoacuity and eye alignment in previously treated amblyopic patients with and without strabismus.

## Methods

The research protocol was approved by the Clinical Research Ethics Committee of the Hospital Universitari Mútua de Terrassa (EO-INT-1912) and was conducted in accordance with the Guide to Good Clinical Practices for Clinical Trials and conforms to the precepts and guidelines of the Declaration of Helsinki on the ethical principles of biomedical research in humans. Written informed consent was obtained from the parents or guardians of the study participants and from the participants themselves. The protocol is available at http://www.clincaltrials.gov (identifier: NCT04315649 [03/16/2020]).

### Participants

Children 5 to 12 years of age who were previously diagnosed with strabismic or anisometropic amblyopia by pediatric eye specialists were eligible. 24 participants were recruited between 16 September and 16 December 2021. All participants in the amblyopic group had completed treatment with spectacles and/or patching or optical penalization treatment but had residual amblyopia. All the participants were diagnosed and treated at the optometric and ophthalmological consultation of the Hospital Universitari Mútua de Terrassa and were subsequently evaluated and recruited by the Optometry department of the same hospital. Inclusion criteria were: (1) prior diagnosis of strabismic and/or refractive amblyopia, (2) present a residual amblyopia defined as best-corrected visual acuity (BCVA) ≥0.10 logMAR in one or both eyes and interocular difference in BCVA ≥0.10, (3) amblyopia treatment completed at least 6 months before the intervention, (4) stable VA during at least 9 months, (5) absence of associated ocular pathologies, (6) not having seen a 3D movie before. In addition to the general criteria, the following inclusion criteria were taken into consideration for the group with strabismus: (1) present strabismus (2) angle of deviation equal or smaller than 35 PD; and for the group with anisometropic amblyopia: (1) refractive difference of one diopter or more in the spherical or cylindrical component. Neurotypical children from 5 to 12 years of age were also recruited. The inclusion criteria for this group were: (1) age between 5 and 12 years, (2) absence of amblyopia, (3) absence of other ocular pathologies, (4) not having seen a 3D movie before.

Before the initial evaluation, all participants underwent a comprehensive visual examination that included: retinoscopy and subjective refraction, best corrected visual acuity, near and far vision cover test, near point of convergence, evaluation of extrinsic and intrinsic motility, refractive evaluation under the effects of the cycloplegia, and macular and papillary examination by optical coherence tomography reviewed by an ophthalmologist.

During the experiment, participants used their optical correction, previously reviewed and modified, if necessary, in the prior full visual examination. Patients with strabismus did not wear prisms. All participants wore glasses with polarized lenses provided by the cinema to watch the movie in 3D, and were seated according to their preference by their parents and relatives (S1 Fig).

### Study design

This study was a prospective, pre-post intervention, quasi-experimental pilot study with three groups. The objective of the study was to characterize the change in stereopsis, monocular visual acuity and ocular deviation after watching the 3D movie Sing 2 in a cinema in children with a residual anisometropic or strabismic amblyopia and neurotypical children. The movie was selected because it was one of the Child-Friendly 3D movies of the moment, and able to interest and capture the attention of our participants considering their age range. In addition, the degree of satisfaction of the participants at the end of the film was assessed to ensure that it had aroused their interest and that, therefore, their degree of motivation and attention had been adequate. The screening took place at the specially arranged facilities of the Cines Yelmo in Sant Cugat del Vallès (Barcelona) on March 19, 2022. It should be noted that none of the participants had seen a 3D movie before the intervention, and neither did they watch any 3D movies in the three months after the intervention. No other treatment for amblyopia was carried out during this period.

### Outcome measures

The optometric tests were carried out by six experienced pediatric optometrists in a space equipped with the necessary materials for the evaluation. The visual examinations were carried out in two different rooms with the same instrumentation and matching measurement conditions, paying special attention to lighting conditions and the equipment, in order to unify the examination conditions.

**Fig 1:**
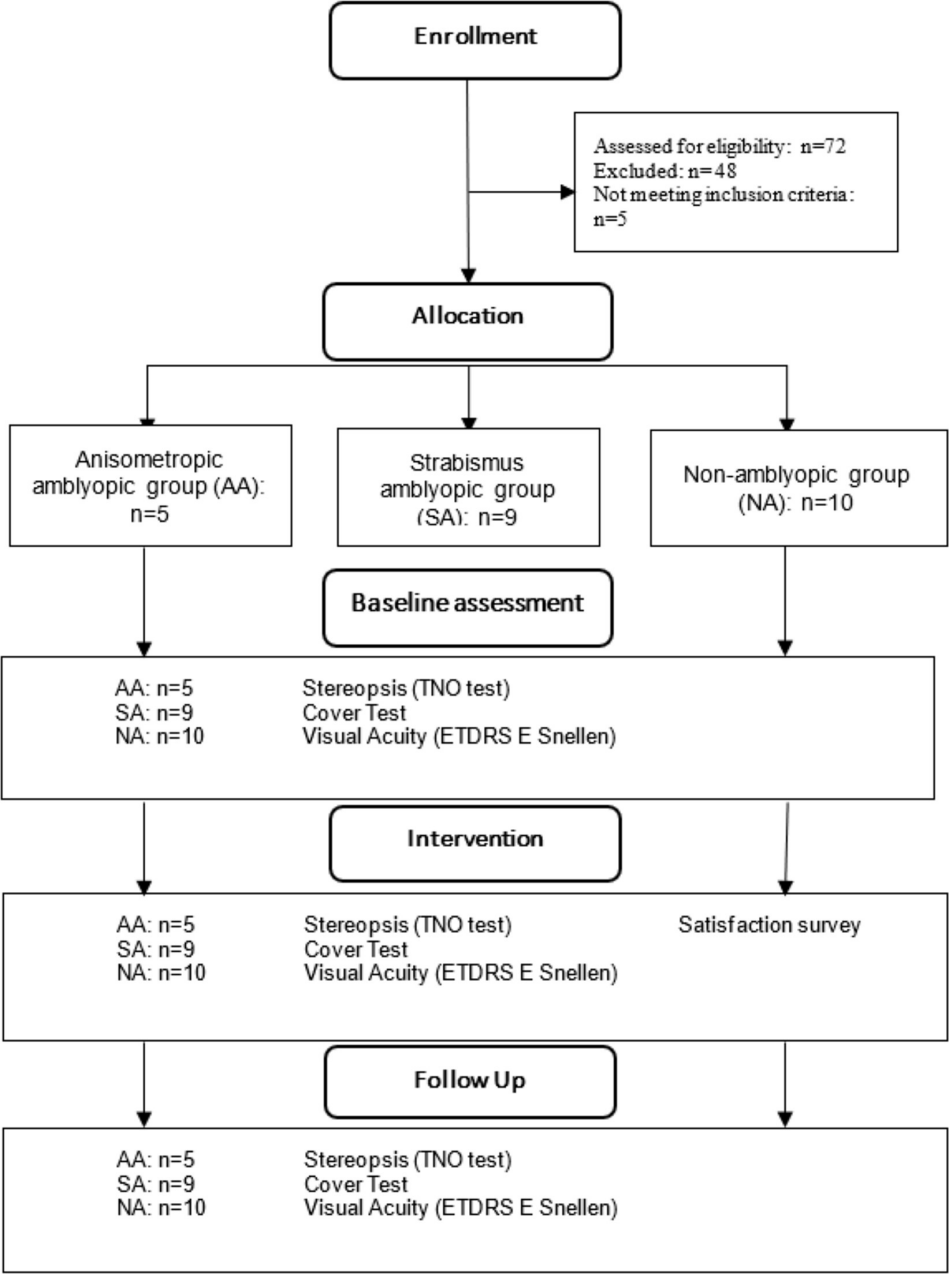
CONSORT diagram.

Visual acuity was measured one week before viewing the movie and 3 months later in the clinic by an expert examiner blinded for the purpose of study, intervention, and characteristics and group of each participant. Stereoacuity and ocular deviation were assessed immediately before and after viewing the movie in the cinema, and three months later in the clinic (Fig 1). Participant was assessed by the same examiner at the screenings in the cinema, who was blind to the characteristics of the participant group, and assessed by the blinded examiner in the clinic. Stereopsis was evaluated with the TNO test. Monocular visual acuity was measured using the Snellen ETDRS E Optotype at 3m. The magnitude of the deviation was determined with prism bars during the cover test at near (40 cm) and distance (6 m). The participants’ degree of satisfaction after the intervention was also evaluated using an ordinal questionnaire (from 1 to 5, 1 being the least and 5 the most satisfaction) based on the Medication Treatment Satisfaction Questionnaire (TSQM) version 1.4 [29].

### Statistical analysis

Data distribution was analyzed using the Shapiro–Wilk test. Mean and standard deviation SD were used to describe parametric data and median and interquartile range (IR) for non-parametric data. Paired t-tests were used to compare means in parametric data, and Wilcoxon’s rank test in non-parametric data. Clinical stereoacuity values in arc sec were transformed to log units for the statistical analysis. The change in outcome variables from baseline to three months after the intervention was analyzed using repeated measures ANOVA (RANOVA) tests. The sphericity of the test was considered using Mauchly’s test, and pertinent corrections were applied in the case of non-assumption of sphericity using the Greenhouse–Geisser correction. SPSS version 27 for Windows was used for the investigation.

## Results

A total of 24 participants were included in the study. The participants’ mean ± SD age was 8.14 ± 2.14 years and 10 were female (41.7%). Fourteen participants were included in the amblyopic group: 9 in the strabismic amblyopia (SA) group, 5 in the anisometropic amblyopia (AA) group; and 10 typically developing children were included in the neurotypical group (Table 1). There were no statistically significant differences between groups in demographic measures at baseline. All participants with a history of amblyopia reported prior amblyopia treatment: 11 children were treated with refractive correction and occlusion (78.5%), 2 participants with refractive correction only (14.3%), and 1 participant with Bangerter filters (7.2%). Individual participants’ details are reported in S1A Table.

**Table 1:** Demographic data (mean age and range).

	Groups			All participants
	Strabismic	Anisometropic	Neurotypical	
Number	9	5	10	24
Age (years)	7.61 [5–11]	7.86 [5–10]	8.94 [6–12]	8.14 [5–12]

**Fig 2:**
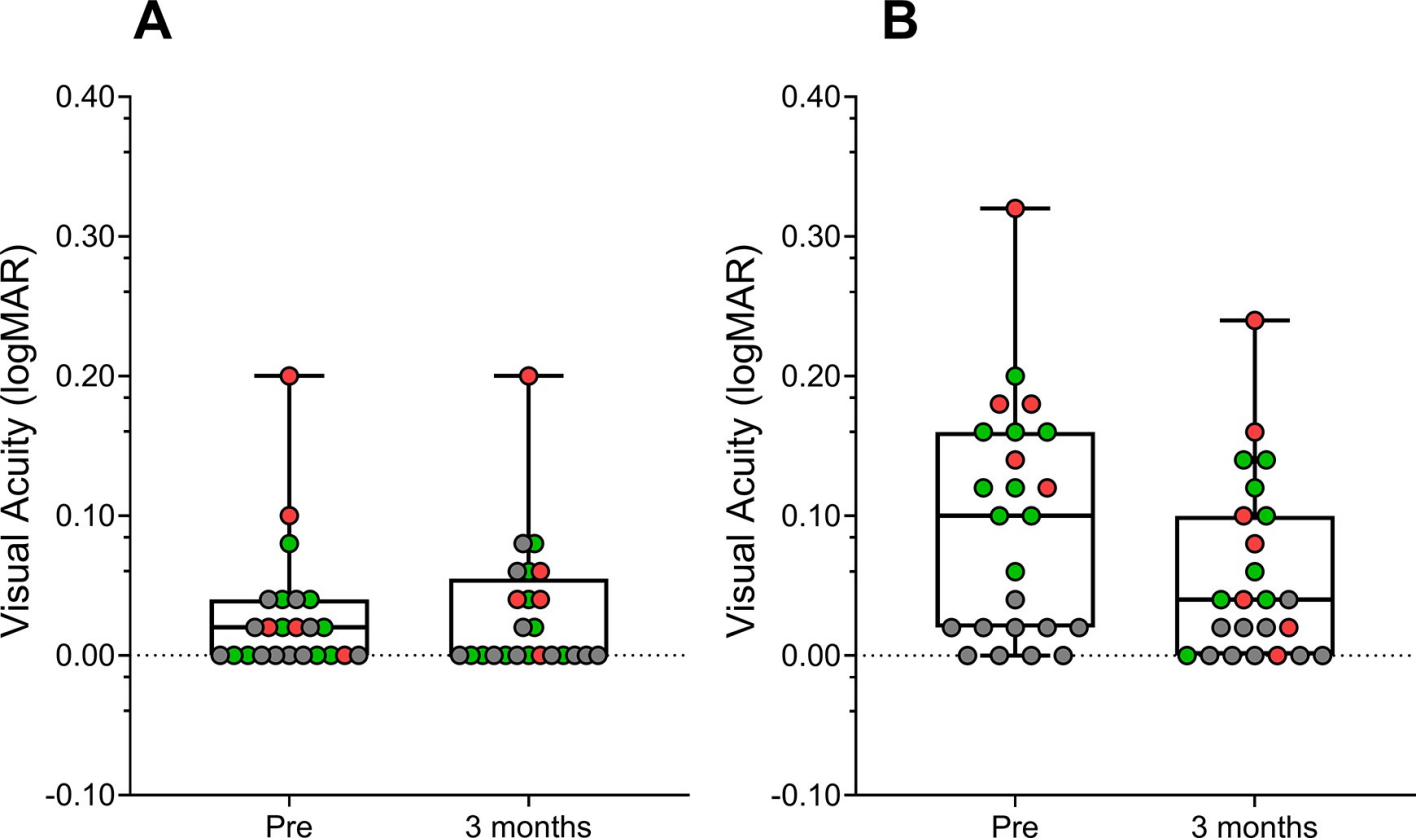
Change in visual acuity before, and 3 months later, after watching the 3D movie of: (A) the dominant eye of the amblyopic group and right eye in the neurotypical group, (B) the non-dominant eye in the amblyopic group and left eye in the neurotypical group. Individual data points are shown as colored bullets, red for the strabismic group, green for the anisometropic group, and gray for the neurotypical group.

One week before watching the movie, median [Interquartile Range (IQR)] visual acuity of the dominant eye in the amblyopia group was 0.02 [0.00–0.05] and mean ± SD of 0.14 ± 0.07 logMAR in the non-dominant (amblyopic) eye. Three months after watching the movie, the visual acuities were 0.03 [0.00–0.06] logMAR and 0.09 ± 0.06 logMAR in the dominant and non-dominant eyes, respectively. Analysis using the t-test for related samples showed a statistically significant change in the non-dominant eye, t(13) = 5.30, P<0.001, IC 95% 0.04 to 0.08, Cohen’s d = 0.953. The dominant eye showed a non-significant improvement between “pre” and “post” intervention measures according to the Wilcoxon’s rank test (P = 1.00). Fig 2 shows the visual acuity results of the amblyopic groups.

In the neurotypical group, right eye visual acuity one week before watching the movie was 0.00 [0.00–0.02] logMAR and 0.00 [0.00–0.03] logMAR 3 months after the intervention (Wilcoxon’s Rrank Test, P = 0.48). Similarly, the left eye visual acuity before watching the movie was 0.02 [0.00–0.02] logMAR and 0.01 [0.00–0.02] logMAR 3 months after the intervention (Wilcoxon’s rank Test, P = 0.32). Visual acuity values are summarized in Table 2.

**Table 2:** Descriptive statistics (mean ± standard deviation for the non-dominant eye in the amblyopia group, and median and interquartile range for the neurotypical group and the dominant eye in the amblyopia group) of visual acuity (VA) in the two groups in logMAR units.

Group	VA Pre	VA 3 months	P-value
Amblyopia	Dom = 0.02 [0.00–0.05]	Dom = 0.03 [0.00–0.06]	P = 1.00
(n = 14)	Non-Dom = 0.15 ± 0.06	Non-Dom = 0.09 ± 0.07	P<0.001
Neurotypical	RE = 0.00 [0.00–0.02]	RE = 0.00 [0.00–0.03]	P = 0.48
(n = 10)	LE = 0.02 [0.00–0.02]	LE = 0.01 [0.00–0.02]	P = 0.32

Dom, Dominant eye; Non-Dom, Non-Dominant eye; RE, right eye; LE, left eye.

The stereoacuity results are summarized in Table 3. Overall, the stereoacuity in the amblyopic group immediately before watching the movie was 2.29 ± 0.32 log arc sec (95% IC, 1,99 to 2,56) (195 arc sec), it was 2.12 ± 0.39 log arc sec (95% IC, 1,75 to 2,46) (131.8 arc sec) right after watching the movie, and 2.07 ± 0.40 log arc sec (95% IC, 1,73 to 2,43) (117.5 arc sec) after three months, which represents an improvement of around 40%. A RANOVA test revealed a statistical significant change in stereoacuity, F(1.25, 16.27) = 9.75, P = 0.01, η_p_^2^ = 0.429.

**Table 3:** Descriptive statistics (mean ± standard deviation) of stereoacuity (ST) in log arc sec in the two groups.

Group	ST Pre	ST Post	ST 3 months	P-value
Amblyopia (n = 14)	2.29 ± 0.32	2.12 ± 0.39	2.07 ± 0.40	P = 0.01
Neurotypical (n = 10)	1.92 ± 0.21	1.80 ± 0.33	1.71 ± 0.37	P = 0.04

**Fig 3:**
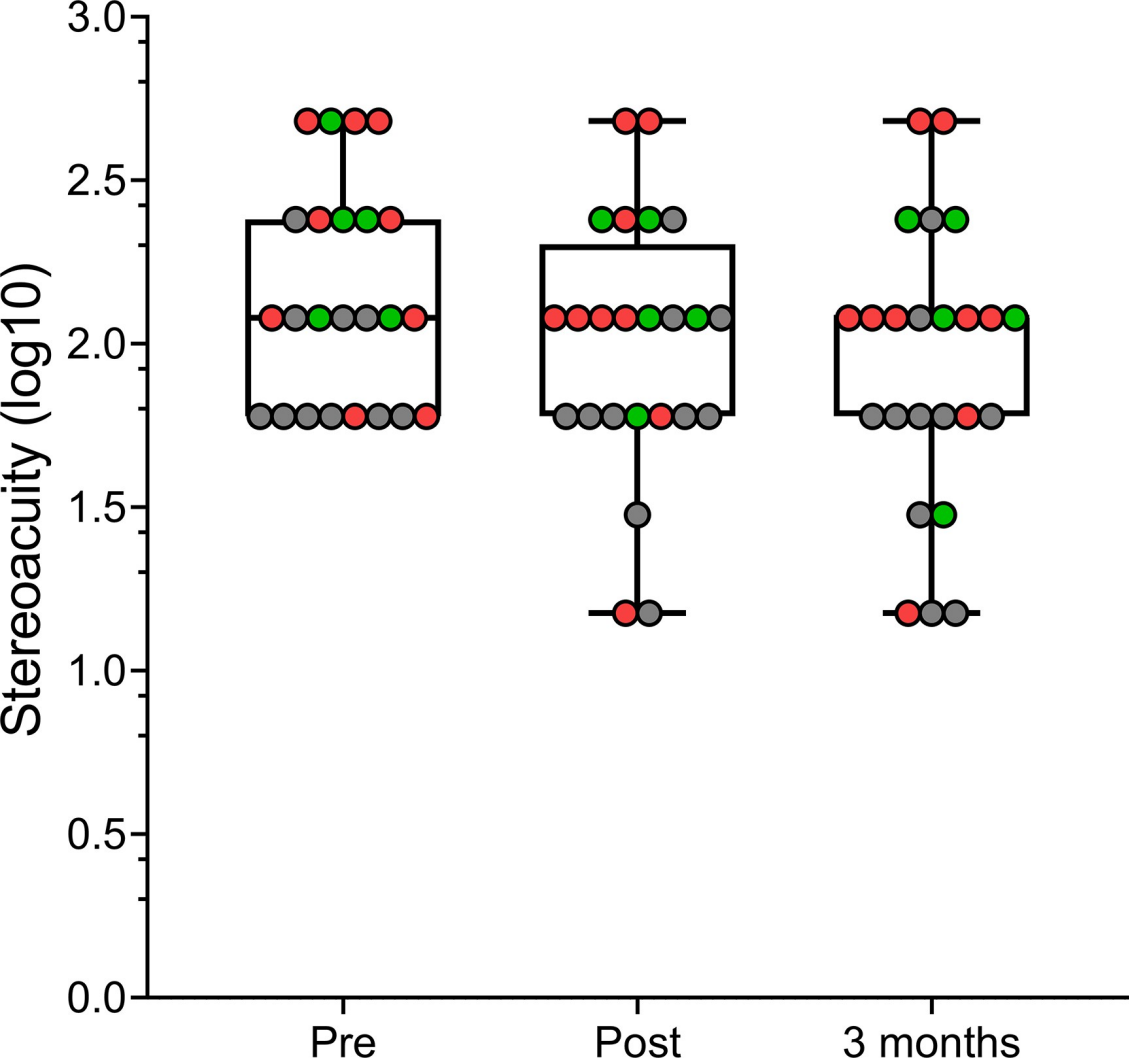
Stereoacuity (log arc sec) in the strabismic group (red), anisometropic group (green) and neurotypical group (gray) immediately before and after watching the 3D movie, and 3 months after the intervention. Individual data points are shown as colored bullets, red for the strabismic group, green for the anisometropic group, and gray for the neurotypical group.

As previous studies have shown that recovery of stereoacuity is more challenging in amblyopic patients with strabismus than in those with anisometropia [30], we compared the change in stereoacuity of the strabismic (n = 9) and anisometropic (n = 5) groups. A repeated measures ANOVA revealed no statistically significant interaction between time and group, F(2,24) = 0.06, P = 0.93, η_p_^2^ = 0.006, suggesting that the change in stereoacuity was similar in the strabismic and anisometropic amblyopia groups (Fig 3). The pre-post intervention change in stereoacuity was of 0.17 ± 0.07 log arc sec in the strabismic group and of 0.18 ± 0.7 log arc sec in the anisometropic group. The mean total change in the strabismic group after three months was 2.07± 0.45 log arc sec, and 2.07± 0.36 log arc sec in the anisometropic group. It should be noted that the small sample size and large individual differences in these subgroups might mask any changes.

In the neurotypical group, before watching the movie, the stereoacuity was 1.92 ± 0.21 log arc sec (83.2 arc sec), right after watching the movie it was 1.80 ± 0.33 log arc sec (63 arc sec), and 1.71 ± 0.37 log arc sec (51.3 arc sec) 3 months later, which represents an improvement of around 38%. A RANOVA test revealed a statistical significant change in stereoacuity, F(2,18) = 4.01, P = 0.04, η_p_^2^ = 0.308.

We evaluated eye alignment using the Cover Test. Exo-deviations (Base-In prism) were represented as negative values, and eso-deviations (Base-Out prism) as positive values. Mean distance ocular deviation for the whole amblyopia group before viewing the 3D movie was -0.07 ± 12.74 prism diopter (PD), range from -35 to 20 PD, 0.38 ± 12.08 PD immediately after the movide, range from -30 to 20 PD, and 0.30 ± 13.31 PD 3 months after the intervention, range from -35 to 25 PD. The change in far ocular deviation was not statistically significant in the amblyopic group, F(2,26) = 0.35, P = 0.70, η_p_^2^ = 0.027, in contrast to the experience reported by other authors. The short exposure time and the small sample size in our study may not have been sufficient to detect changes in the angle of deviation, if they occurred.

**Fig 4:**
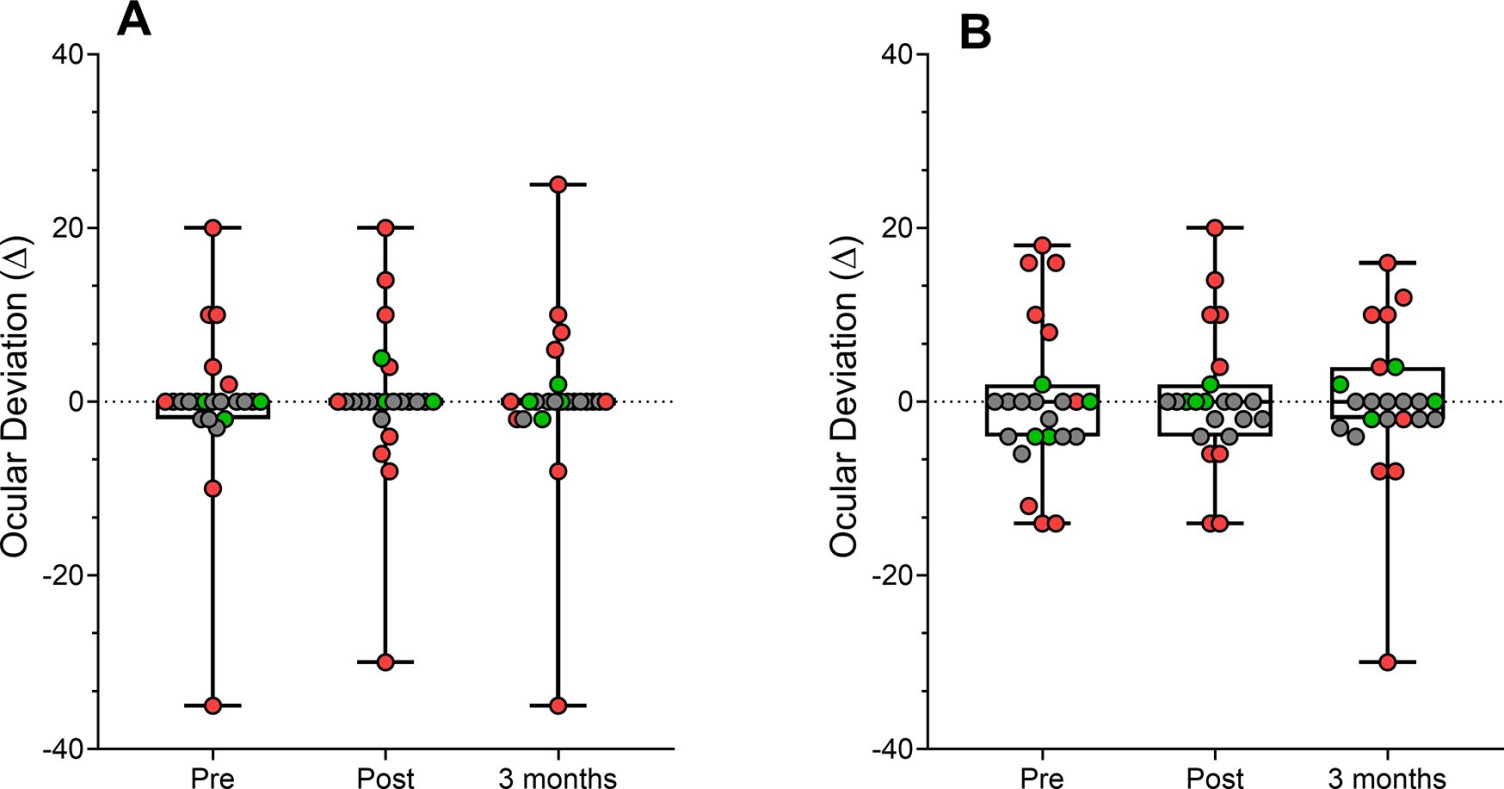
Changes in ocular deviation in prism diopters (PD) for the strabismic group (red), anisometropic group (green) and neurotypical group (grey) at distance and near viewing. Negative values indicate exo-deviation.

There was no statistical significant effect in the factors*group interaction between strabismic and anisometropic groups, F(2,24) = 0.61, P = 0.45, η_p_^2^ = 0.049. Mean near ocular deviation pre-intervention was 1.69 ± 11.31 PD, range from -14 to 18 PD; 1.54 ± 10.20 PD, range from -14 to 20 PD post-intervention, and 0.61 ± 11.79 PD, range from -30 to 16 PD, 3 months after the intervention. Similar statistical results were found with no significant change in ocular deviation at near considering the overall amblyopic group, F(2,24) = 0.059, P = 0.94, η_p_^2^ = 0.005, and between strabismic and anisometropic groups, F(2,24) = 1.94, P = 0.17, η_p_^2^ = 0.139. Fig 4 shows the individual changes in the ocular deviation in the amblyopic group and neurotypical group in far and near ocular deviation.

Sensory function and its recovery in strabismus differs between divergent and convergent deviations, constant and intermittent deviations, and depends on the magnitude of the deviation. We compared the change in ocular deviation of participants with exotropia (n = 6) and esotropia (n = 7) in the amblyopic group. A repeated measures ANOVA revealed no statistically significant interaction between time and group in mean distance ocular deviation, F(2,22) = 0.03, P = 0.97, η_p_^2^ = 0.003, and no statistically significant interaction between time and group in near ocular deviation, F(2,22) = 1.07, P = 0.36, η_p_^2^ = 0.089, suggesting that there were no differences between the variation in ocular deviation between exo- and eso-deviations.

In the neurotypical group, mean values in far ocular deviation before viewing the movie were -0.70 ± 1.16 PD, range from -3.00 to 0 PD, -0.20 ± 0.63 PD, range from -2.00 to 0 PD, immediately after viewing the movie, and -0.20 ± 0.63 PD, range from -2 to 0 PD 3 months after the intervention. Near ocular deviation were -2.00 ± 2.30 PD, range from -6.00 to 0 PD, in “pre”, -1.40 ± 1.64 PD, range from -4.00 to 0 PD, in “post”, and -1.30 ± 1.49 PD, range from -4.00 to 0 PD after 3 months. No statistically significant changes were found in the phoria values in both far and near viewing conditions (P>0.05).

Twenty-three participants completed a questionnaire (1 dropout). 91.3% of the participants indicated that they enjoyed the movie very much (S1B Table) and 97,65% of the participants indicated that they would watch a 3D movie again, even those participants who reported some discomfort while watching the movie (21.7%). Participants in the amblyopic group had more ocular symptoms compared to the control group. Some of the most frequently reported complaints were: head ache, ocular pain, and blurred vision. This suggests that this new approach might be well accepted by the participants and their families. No statistically significant differences were observed between the responses of the three study groups.

## Discussion

The objective of this pilot study was to determine whether watching a 3D movie with large disparities might be a useful tool to improve stereoacuity. Specifically, we set out to ask whether Bridgeman’s hypothesis that *"sustained attention to various high-disparity stereoscopic challenges in an engaging immersive environment"* might have led to such a marked improvement in their stereoacuity.

For our study, we recruited 5 participants with anisometropic amblyopia, 9 participants with strabismic amblyopia and 10 neurotypical children and invited them to watch the 3D movie "Sing 2" in a cinema. We chose this movie because it is child-friendly and would capture the attention and interest of our participants considering their age range. Moreover, the large field of view, between 41.4 and 74.2 visual degrees, also enables the presentation of large binocular disparities, which may be important for improving stereovision in both neurotypical adults [28] and in patients with amblyopia [26, 31, 32].

Our results are in accordance with previous studies [20, 21, 23, 26, 31, 33], showing that most of the stereo anomalous participants improved their prior stereopsis after viewing the 3D movie, and, more importantly, this improvement was maintained after three months (Fig 3). It is known that stereoacuity can be improved through perceptual learning in normal adults as well as in strabismic amblyopic adults [13, 21, 24]. In addition, in recent years, treatments for amblyopia based on action video games have been developed to increase compliance and treatment efficacy. These appear to effectively improve visual acuity [25, 34, 35] and, recently, they have been demonstrated to improve stereovision in adults with amblyopia playing in a virtual reality environment [26, 31, 33], (See Levi, 2020; 2022, 2023 for recent reviews) [17, 18, 32].

However, unlike the approach presented in the current study, these interventions required a longer training time to achieve improvements in stereovision. For example, Li et al. (2018) showed a 0.11 LogMAR improvement (≈ 29%) in visual acuity and a mean 34% improvement in stereopsis in 21 adults with amblyopia after playing action video games for 40 hours in an immersive 3D environment [26]. Vedamurthy et al. (2016) showed improvements in stereoacuity of up to 30% in adults with strabismic amblyopia after 40 hours of training in a visuomotor task in a virtual reality environment, with no evidence of changes in visual acuity [33]. In this study, we found significant improvements in stereoacuity in participants with residual anisometropic and strabismic amblyopia with a single 1-hour and 50-minute film session. The differences in time required could be due to the age of the participants and/or their intrinsic characteristics. Future studies in older participants could be interesting to conduct. In addition, our sample included both strabismic and anisometropic amblyopia, unlike some other studies [33], and the sample of strabismic participants also contained non-constant or intermittent strabismus. This difference could be also explained by the approach itself, mainly the use of an immersive environment with large disparities, as is the case when watching a 3D movie in a cinema.

Binocular perception is altered in amblyopia primarily due to suppression, which results in degraded sensory fusion and poor or absent stereopsis [30]. Our small sample size of participants with anisometropia (n = 4) and strabismus (n = 9) did not allow us to make definite statistical inferences beyond the neurotypical and amblyopic groups. However, it is well known that the reduction in stereoacuity is less severe in anisometropic amblyopia than in the presence of strabismus [30]. In addition, it is well documented in the literature that individuals with anisometropia are more likely to recover stereopsis after amblyopia treatment than individuals with strabismus [21, 30]. We observed that some strabismic amblyopic participants showed no improvement in stereo acuity tests. Specifically, participants S7 and S8 (S1A Table), both with constant and large-angle strabismus. This raises the question of whether our approach is suitable for patients with constant large-angle strabismus, or whether it might be helpful to include an image rebalancing system or to increase the exposure time to treatment. It also raises the question for future investigation about the need to use of prisms and or surgery to achieve ocular alignment mechanisms for patients with strabismus, as other authors have suggested in their protocol [31].

Previous studies have shown a correlation between visual acuity and stereopsis in amblyopia, with differences observed between the two subtypes of amblyopia [30]. Our results suggest that there may be a relationship between improvement in visual acuity and improvement in stereoacuity as opposed to other results where improvements in stereopsis were observed independently of visual acuity [21].

In reference to the neurotypical group, our results also show improvements in stereoacuity (P = 0.04). Our sample includes participants with low ST values, especially those participants under 9 years of age [36, 37]. Other studies have found a gradual improvement in stereoacuity scores with increasing age, and normal stereo acuity of 40 arcsec was discovered at 9 years of age. While previous studies have shown that prolonged perceptual learning or 3D videogame play can improve stereopsis in neurotypical adults [22, 28], the current study showed a significant improvement in stereoacuity in stereo normal children after watching a 3D movie for less than 2 hours. It should be noted that, although the current study included a neurotypical group, this is not an active control group.

Our pilot study has several limitations. These include the small sample, and the absence of an active control group viewing the same movie in 2D. Power analysis using G*Power [38], a free-available software able to calculate a required sample size using input parameters depending on the statistical test (i.e. difference between two independent means), revealed that the analysis of change in visual acuity in the amblyopic group achieved a statistical power of 82.15%, and 58.56% for stereoacuity in this study.

Playing 2D videogames results in improved stereopsis in some adults with anisometropic amblyopia [39]. Consequently, without a 2D control group we cannot conclusively attribute the improvements to the presence of large disparities. Could the improvements in both visual acuity and stereoacuity be due to practice (perceptual learning) effects? We argue that this is unlikely to account for the improvement in VA for several reasons: first, the amblyopia group had been tested on several occasions and had stable visual acuity for at least 9 months; moreover, the mean difference in VA between test and retest in normal and amblyopic children under the age of 12 was 0.01 LogMar [40]. On the other hand, clinical stereotests such as the TNO test used here have poor test-retest repeatability in those with abnormal binocular vision. For example, Antona et al. [10] reported a coefficient of repeatability of ±54 arc sec in their neurotypical group and 120 arc sec in individuals with abnormal binocular vision. However, it should be noted that, while in our study some participants improved, others regressed, so that the group mean difference was just -5 and -3 arc sec in our neurotypical and amblyopic groups respectively, much smaller than the ≈ 32 arc sec improvement in our neurotypical group and 77.5 arc sec improvement in our amblyopic group.

To summarize, our pilot study showed the possibility of having a short-duration and attractive approach to improve stereovision in amblyopic and neurotypical children, providing evidence that watching 3D movies with large disparities in a cinema could be an important addition to the armamentarium for improving stereopsis in both amblyopic and neurotypical patients aged 5 to 12 years. In addition, we found an improvement in visual acuity of the amblyopic eye, which was maintained 3 months after the intervention. Therefore, our results suggest that watching 3D movies with large disparities in a rich and immersive environment may promote improvement in visual acuity and stereovision in children with residual strabismic or anisometropic amblyopia in a relatively short time.

This pilot study shows the clinical potential application of viewing 3D movies in real cinema for improving visual functions in amblyopia. Future clinical trials including a larger sample size, different ages of participants, a control group that views 2D movies, and more 3D movies to watch to see the time-dependent effect would be interesting to conduct.

## Supporting information

S1 Checklist TREND statement checklist. (PDF)

S1 Fig Seating of participants in the cinema. (DOCX)

S1 Table **A.** Participant characteristics. **B.** Questionnaire results.
(ZIP)

S1 File (PDF)

S2 File (PDF)
