# Supplementary material for: Can viewing a 3D movie improve visual function in children with a history of amblyopia and neurotypical children?: A pilot study
Source: PLoS One. 2024 Jun 25;19(6):e0305401. doi: 10.1371/journal.pone.0305401 (PMC11198783; doi:10.1371/journal.pone.0305401)
Supplement: S1 Fig — (DOCX) [file pone.0305401.s002.docx]

**Fig S1. Seating of participants in the cinema.** The green circle corresponds to participants with strabismic amblyopia, the yellow circle to participants with anisometropic amblyopia and the blue circle to the neurotypical group.
