# Supplementary material for: Can viewing a 3D movie improve visual function in children with a history of amblyopia and neurotypical children?: A pilot study
Source: PLoS One. 2024 Jun 25;19(6):e0305401. doi: 10.1371/journal.pone.0305401 (PMC11198783; doi:10.1371/journal.pone.0305401)
Supplement: S1 File — (PDF) [file pone.0305401.s004.pdf]

VISUALITZACIÓ DE PEL·LÍCULES 3D PER A LA RECUPERACIÓ DE LA FUNCIÓ VISUAL EN NENS AMB AMBLIOPIA RESIDUAL I NENS SENSE AMBLIOPIA

PROTOCOL DE RECERCA

NOVEMBRE 2020

RESUM

L'objectiu d'aquest estudi és avaluar si la visualització d'una pel·lícula en 3D immersiva amb grans disparitats en un cinema dona com a resultat una millora de la percepció de profunditat estereoscòpica, desviació ocular i agudesa visual en 24 nens de 5 a 12 anys amb ambliopia anisometròpica i/o estrabística prèviament tractats. amb oclusió i nens neurotípics sense ambliopia. Les mesures es faran abans de veure la pel·lícula, immediatament després i tres mesos després. La nostra hipòtesi és que la visualització de pel·lícules en 3D amb grans disparitats tindrà un efecte de millora de l''estereopsis, l'agudesa visual de l'ull no dominant i la desviació ocular en nens ambliops de 5 a 12 anys tractats prèviament.

RESUM 2

INFORMACIÓ GENERAL 6

JUSTIFICACIÓ 7

REFERENCES 8

OBJECTIU GENERAL I ESPECÍFICS 9

OBJECTIU GENERAL 9

OBJECTIUS ESPECÍFICS 9

DISSENY D'ESTUDI 9

METODOLOGIA 10

CONSIDERACIONS DE SEGURETAT 11

SEGUIMENT 12

GESTIÓ DE DADES I ANÀLISI ESTADÍSTICA 12

GARANTIA DE QUALITAT 12

RESULTATS ESPERATS DE L'ESTUDI 13

DIFUSIÓ DE RESULTATS I POLÍTICA DE PUBLICACIONS 13

GESTIÓ DE PROJECTES 14

ÈTICA 14

CONSENTIMENT INFORMAT I FULL INFORMACIÓ 15

FULL INFORMACIÓ 15

CONSENTIMENT INFORMAT **16**

PRESSUPOST **19**

CURRÍCULUM VITAE DELS INVESTIGADORS **19**

SUPORT AL PROJECTE I FINANÇAMENT **20**

INFORMACIÓ GENERAL

Títol: Visualització de pel·lícules en 3D per a la recuperació de la funció en nens amb ambliopia residual i nens sense ambliopia.

Noms dels investigadors: Laura Asensio-Jurado[1,2,3], Marc Argilés[1,2], Lluïsa Quevedo-Junyent[2], Clara Mestre[1], Dennis M. Levi[4]

Afiliacions:(1) Centre for Sensors, Instruments and Systems Development (CD6), Universitat Politècnica de Catalunya, Terrassa, Spain, (2) Departament d'Òptica i Optometria, Universitat Politècnica de Catalunya BarcelonaTech (UPC), Terrassa, Spain, (3) Hospital Universitari MutuaTerrassa, Terrassa, Spain, (4) Herbert Wertheim School of Optometry & Vision Science, University of California, Berkeley., CA, United States.

Informació de contacte: Laura Asensio-Jurado, laura.asensio@upc.edu

Número telèfon de contacte: +34636760450

JUSTIFICACIÓ

L'ambliopia és un trastorn del neurodesenvolupament visual associat, amb més freqüència, a la presència d'estrabisme i anisometropia. És la condició visual clínicament més important en la infància perquè, a part de l'error de refracció, és la causa més freqüent de pèrdua de visió en nens. És una afecció reversible que afecta fins a un 2,9%[1] de la població, i és el resultat d'una experiència visual anòrmala durant el període més sensible del desenvolupament visual. Encara que l'ambliopia s'expressa en la pràctica clínica com una reducció de l'agudesa visual, també es caracteritza per una estereoscòpia alterada. Generalment, els tractaments d'ambliopia se centren en la recuperació de l'agudesa visual, i no hi ha molts tractaments que se centren principalment en la recuperació de l'estereopsis, tan present i amb un impacte funcional important. S'han desenvolupat nous enfocaments que busquen restaurar la visió binocular i se centren en la rehabilitació de l'estereopsis en persones amb ambliopia i/o estrabisme amb resultats encoratjadors. Aquests nous enfocaments inclouen l'aprenentatge perceptiu[2-4], els videojocs d'acció[5-7] i la visualització de pel·lícules en 3D[8]. L'objectiu d'aquest estudi és revisar, analitzar i, si escau, actualitzar el model de tractament actual de l'ambliopia. D'una banda, avaluar l'efectivitat de la visualització d'una pel·lícula en 3D en la millora de l'estereoagudesa i la desviació ocular, així com l'agudesa visual. D'altra banda, avaluar quantitativament el grau de satisfacció i acceptació dels participants i les seves famílies envers la intervenció.

REFERENCES

1 Fu, Z. *et al.* Global prevalence of amblyopia and disease burden projections through 2040: a systematic review and meta-analysis. *Br J Ophthalmol* 104, 1164-1170, doi:https://10.1136/bjophthalmol-2019-314759 (2020).

2 Li, R. W. *et al.* Sharpening coarse-to-fine stereo vision by perceptual learning: asymmetric transfer across the spatial frequency spectrum. *R Soc Open Sci* 3, 150523, doi:https://10.1098/rsos.150523 (2016).

3 Ding, J. & Levi, D. M. Recovery of stereopsis through perceptual learning in human adults with abnormal binocular vision. *Proc Natl Acad Sci U S A* 108, E733-741, doi:https://10.1073/pnas.1105183108 (2011).

4 Portela-Camino, J. A., Martín-González, S., Ruiz-Alcocer, J., Illarramendi-Mendicute, I. & Garrido-Mercado, R. A Random Dot Computer Video Game Improves Stereopsis. *Optom Vis Sci* 95, 523-535, doi:https://10.1097/opx.0000000000001222 (2018).

5 Levi, D. & Li, R. W. Playing 3-dimensional (3D), but not 2D video games can improve stereoacuity in neurotypical observers. *Journal of Vision* 19, 130a-130a, doi:https://10.1167/19.10.130a (2019).

6 Godinez, A., Martín-González, S., Ibarrondo, O. & Levi, D. M. Scaffolding depth cues and perceptual learning in VR to train stereovision: a proof of concept pilot study. *Sci Rep* 11, 10129, doi:10.1038/s41598-021-89064-z (2021).

7 Vedamurthy, I. *et al.* Recovering stereo vision by squashing virtual bugs in a virtual reality environment. *Philos Trans R Soc Lond B Biol Sci* 371, doi:https://10.1098/rstb.2015.0264 (2016).

8 Li, R. W. *et al.* Improving Adult Amblyopic Vision with Stereoscopic 3-Dimensional Video Games. *Ophthalmology* 125, 1660-1662, doi:https://10.1016/j.ophtha.2018.04.025 (2018).

OBJECTIU GENERAL I ESPECÍFICS

Objectiu general

- Estudiar l'efecte sobre la funció visual de la visualització d'una pel·lícula 3D en pacients ambliops ambliòpics i/o anisometròpics residuals d'entre 5 i 12 anys.

Objectius específics

- Avaluar l'efecte de la visualització d'una pel·lícula en 3D sobre l'estereopsis, l'agudesa visual i la desviació ocular.
- Establir possibles correlacions entre les diferents variables.
- Comparar l'efecte d'aquesta intervenció per als subtipus d'ambliopia refractiva i estràbica.
- Aportar dades sobre el grau de satisfacció i sentiment de pares i fills en relació a la intervenció proposada.

DISSENY D'ESTUDI

Es realitzarà un estudi quasi experimental, sense grup control, pre i post-intervenció en el qual es seleccionaran subjectes amb ambliopia residual refractiva i/o estrabisme, que hagin estat sotmesos al tractament tradicional de l'ambliopia. Les variables d'estereopsis, desviació latent o manifesta i agudesa visual s'avaluaran abans de veure la pel·lícula, immediatament després i tres mesos després. La mostra inclourà subjectes d'entre 5 i 12 anys amb diagnòstic d'ambliopia refractiva i/o estrabística residual. Tots els participants han seguit un tractament convencional per a l'ambliopia, l'oclusió o el tractament de penalització, abans de participar en l'estudi.

Els criteris d'inclusió seran: (1) diagnòstic previ d'ambliopia estrabica i/o anisometropica, (2) presentar ambliopia residual definida com l'agudesa visual millor corregida (BCVA)

de ≥ 0,10 logMAR en un o ambdós ulls i una diferència interocular ≥0,10, (3) tractament ambliopia completat almenys 6 mesos abans de la intervenció, (4) estabilitat AV superior o igual a 9 mesos, (5) absència de patologies oculars associades, (6) no haver vist una pel·lícula en 3D abans. A més dels criteris generals, es tindran en compte els criteris d'inclusió següents per al grup amb estrabisme: (1) estrabisme present (2) angle de desviació igual o inferior a 35 PD; i per al grup amb ambliopia anisometròpica: (1) diferència de refracció d'una diòptria o més en el component esfèric o cilíndric.

També es reclutaran nens neurotípics de 5 a 12 anys. Els criteris d'inclusió d'aquest grup seran: (1) edat entre 5 i 12 anys, (2) absència d'ambliopia, (3) absència d'altres patologies oculars, (4) no haver vist abans una pel·lícula en 3D.

METODOLOGIA

La present investigació és un estudi d'intervenció prospectiu pre-post experimental en què es seleccionaran participants amb ambliopia residual refractiva i/o estrabística que han seguit prèviament un tractament tradicional per a l'ambliopia. Els subjectes que compleixin els criteris d'inclusió de l'estudi seran informats i signaran el consentiment informat per a la seva inclusió a l'estudi. La mostra inclourà participants d'entre 5 i 12 anys avaluats pel departament d'optometria de l'Hospital Universitari de Terrassa. Els participants es dividiran en tres grups. El primer grup inclourà ambliops estrabístics, el segon grup inclourà ambliops refractius i el tercer grup inclourà nens neurotípics.

Abans de l'avaluació inicial, tots els participants es sotmetran a un examen visual complet que inclourà: agudesa visual no corregida, retinoscòpia i refracció subjectiva, millor agudesa visual corregida, prova de cobertura de visió de prop i llunyà, punt de convergència proper, valoració de la motilitat extrínseca i intrínseca, avaluació de la refracció sota el efectes de l'examen cicloplègic i macular i papil·lar mitjançant tomografia de coherència òptica revisada per un oftalmòleg.

La intervenció consistirà en la visualització d'una pel·lícula en 3D. Aquesta serà seleccionada pel seu disseny 3D, adequat a l'edat dels participants i també, la pel·lícula serà actual i interessant per als participants per tal de garantir la seva atenció i motivació. L'estudi es farà als cinemes Yelmo de Sant Cugat i la pel·lícula encara està per concretar.

Durant la visualització, els participants utilitzaran la seva correcció habitual, prèviament revisada i modificada, si cal, en l'examen visual complet previ. Els pacients amb estrabisme no portaran correcció prismàtica. I, a més, tots els participants portaran les ulleres amb lents polaritzades per veure la pel·lícula en 3D, cedides pel cinema

Per a avaluar l'efecte de veure una pel·lícula en 3D sobre l'estereoagudesa, l'agudesa visual monocular i la desviació ocular s'utilitzaran els teest seguents. L'estereopsis es mesurarà amb la prova TNO. La prova TNO consta de 7 fulls per a l'avaluació estereoscòpica i s'ha d'utilitzar amb ulleres vermelles-verdes. Es mesurarà l'agudesa visual monocular d'ambdós ulls mitjançant l'optotip Snellen ETDRS E a 3 m i la magnitud de la desviació ocular es mesurarà amb varetes de prismes durant la prova de cobertura a prop (40 cm) i distància (6 m). Totes les mesures seràn realitzades per optometristes pediatrics experts

Al final de la intervenció, es valorarà el grau de satisfacció dels participants mitjançant un qüestionari ordinal (de l'1 al 5, sent 1 el menys satisfacció i 5 el més satisfactori) basat en el Qüestionari de satisfacció amb el tractament amb medicaments (TSQM). versió 1.4.

CONSIDERACIONS DE SEGURETAT

Les dades recollides per a l'estudi s'identificaran mitjançant un codi i només els investigadors d'aquest estudi podran enllaçar aquestes dades i la història clínica. Les dades recollides per aquest estudi s'enregistraran únicament mitjançant un codi de manera que no s'inclogui cap informació de cap tipus que permeti la identificació dels

participants. Només l'investigador de l'estudi i els seus col·laboradors amb el seu permís específic podran enllaçar les dades a la història clínica.

La identitat dels participants no estarà disponible per a ningú excepte per una emergència mèdica o requisit legal. Les autoritats sanitàries, el Comitè d'Ètica de la Recerca i el personal autoritzat pel promotor de l'estudi poden tenir accés a la seva informació personalment identificada, quan sigui necessari per comprovar les dades i procediments de l'estudi, però sempre mantenint la confidencialitat d'acord amb la legislació vigent.

Només es transferiran les dades codificades a tercers i a altres països, que en cap cas han de contenir informació que pugui identificar directament el pacient. En el cas que es produeixi aquesta cessió, tindrà la mateixa finalitat que l'estudi descrit i garantint-ne la confidencialitat.

D'acord amb el que disposa l'esmentada legislació, pot exercir els drets d'accés, modificació, oposició i cancel·lació de les dades.

SEGUIMENT

Les mesures es faran abans de veure la pel·lícula, immediatament després i tres mesos després.

GESTIÓ DE DADES I ANÀLISI ESTADÍSTICA

Les variables qualitatives es descriuran amb percentatges i les quantitatives amb la mitjana, la mediana, la desviació estàndard i els valors màxim i mínim. Els valors d'estereoagudesa clínica en segons arc es transformaran en unitats logarítmiques per a l'anàlisi. El canvi en les variables de resultat des de la línia inicial fins als tres mesos després de la intervenció s'analitzaran mitjançant mesures repetides ANOVA (RANOVA). Es considerarà l'esfericitat de la prova mitjançant la prova de Mauchly i s'aplicaran les correccions pertinents. Per realitzar l'anàlisi s’utilitzarà la versió 27 de SPSS per a Windows.

GARANTIA DE QUALITAT

Aquesta investigació segueix i compleix la GCP que garanteix als pacients els seus drets, seguretat i benestar en l'estudi.

El tractament de les dades, la comunicació i la cessió de les dades personals de tots els subjectes participants, es realitzaran d'acord amb el que disposa el Reglament núm. 2016/679 del Parlament Europeu i del Consell, de 27 d'abril de 2016, relatiu a la protecció de les persones físiques pel que fa al tractament de dades personals i a la lliure circulació de dades, i a la Llei orgànica de protecció de dades de caràcter personal, 18 de desembre i 2016.

RESULTATS ESPERATS DE L'ESTUDI

En la consulta optomètrica pediàtrica, l'ambliopia és la condició més important a detectar i tractar per garantir un correcte desenvolupament visual i perceptiu, i així afavorir un correcte aprenentatge i desenvolupament personal. Disposem de protocols de cribratge a l'atenció primària per detectar precoçment aquesta afecció i iniciar el tractament. La teràpia oclusiva passiva ha estat àmpliament estudiada i s'ha demostrat la seva eficàcia en la millora de l'agudesa visual. Malgrat la provada eficàcia en nens, els resultats fracassen en més del 30% dels casos, i els que responen al tractament sovint tenen ambliopia residual. A més, l'oclusió té un alt índex d'incompliment pel fet que és una teràpia a llarg termini i poc atractiva per als nens.

La introducció de noves intervencions en el tractament de l'ambliopia sorgeix de la voluntat d'oferir una alternativa al tractament tradicional. D'una banda, persegueixen l'objectiu de millorar les taxes de temps de resposta al tractament i les taxes de recurrència. I d'altra banda, es pretén oferir una alternativa de tractament més acceptada pels usuaris introduint elements lúdics però alhora incorporant altres rehabilitadors.

Tot i així, resulta molt atractiu proposar que la visualització de pel·lícules en 3D amb determinades característiques tècniques es pugui traduir en millores de les variables psicofísiques alterades en l'ambliopia, i amb una major acceptació entre els infants.

DIFUSIÓ DE RESULTATS I POLÍTICA DE PUBLICACIONS

Els resultats del projecte es difondran en publicacions de referència en revistes i en congressos internacionals i nacionals. A més, els participants de l'estudi i les seves famílies seran informats del resultat de les seves diferents avaluacions, així com dels resultats finals de l'estudi quan estiguin disponibles.

DURACIÓDEL PROJECTE

ESTAPES DE L'ESTUDI	2019		2020												2021												2022											
	NOV	DEC	GEN	FEB	MAR	ABR	MAI	JUN	JUL	AGO	SEP	OCT	NOV	DEC	GEN	FEB	MAR	ABR	MAI	JUN	JUL	AGO	SEP	OCT	NOV	DEC	GEN	FEB	MAR	ABR	MAI	JUN	JUL	AGO	SEP	OCT	NOV	DEC
RECERCA BIBLIOGRÀFICA																																						
CONCEPTUALITZACIÓ DE L'ESTUDI																																						
DISSENY METODOLOGIA																																						
COMITÈ ÉTIC I REGISTTRE ESTUDI																																						
ORGANITZACIÓ I PLANIFICACIÓ																																						
RECLUTAMENT																																						
EXCECUCIÓ																																						
TRACTAMENT I ANÀLISIS DE DADES																																						
REDACCIÓ INFORME																																						

GESTIÓ DE PROJECTES

Laura Asensio Jurado, Marc Argilés i Lluïsa Quevedo Junyent van contribuir a la conceptualització de l'estudi, idear la metodologia. Van formar part de l'equip encarregat de les avaluacions i la recollida de dades. Van dur a terme la preservació i l'anàlisi de dades. Posteriorment i, juntament amb Clara Mestres i Dennis M. Levi, va contribuir a la validació de dades i a la redacció dels resultats.

ÈTICA

L'estudi es realitzarà d'acord amb les Guies de bona pràctica clínica en els assaigs clínics (2016) i els preceptes de la Declaració d'Hèlsinki (Brasil, 2013) sobre principis ètics per a la investigació amb éssers humans.

Els pacients i els seus pares o tutors seran informats de la naturalesa de l'estudi i hauran de donar el seu consentiment informat per escrit, del qual en rebran una còpia signada. Se'ls informarà clarament que poden abandonar l'estudi en qualsevol moment sense que això afecti el seu seguiment o tractament. En l'aspecte econòmic, els implicats en aquest estudi no rebran cap tipus de compensació.

La confidencialitat de les dades dels pacients està garantida d'acord amb el Reglament nº 2016/679 del Parlament Europeu i del Consell, de 27 d'abril de 2016, sobre protecció de dades personals. Es mantindrà la confidencialitat de les dades transferint-les de la història clínica a una base de dades codificada per a l'anàlisi.

CONSENTIMENT INFORMAT I FULL INFORMACIÓ

FULL INFORMACIÓ

VEURE UNA PEL·LÍCULA EN 3D POT MILLORAR LA FUNCIÓ VISUAL EN NENS AMBLIÒPICS I NEUROTÍPICS.

L'estudi clínic pretén avaluar l'efectivitat en la millora visual després de veure una pel·lícula en 3D, així com el grau de satisfacció i interferència en la rutina diària del nen. El tractament que cal dur a terme consisteix a veure una pel·lícula en 3D. Abans i després de la visualització de la pel·lícula, es realitzarà una avaluació visual consistent en la mesura de l'agudesa visual en visió de lluny i de prop, estereopsis (visió 3D) i la mesura de la desviació ocular. A més, al final de la pel·lícula hi haurà un petit qüestionari per avaluar la satisfacció en relació a l'activitat. Al cap de tres mesos, s'oferirà una nova avaluació visual per valorar possibles millores produides per aquest tractament. La pel·lícula ha estat seleccionada amb uns criteris de no-violència molt estrictes, sent adequats a l'edat dels participants.

Per a qualsevol informació o aclariment que necessiteu, no dubteu en contactar amb mi.

Laura Asensio Jurado

ambliopiatt@gmail.com

CONSENTIMENT INFORMAT

VISIONAT DE PEL·LÍCULES 3D COM A EINA EN LA RECUPERACIÓ DE LA ESTEREOPSIS EN AMBLIOPS ANISOMETROPICS I/O ESTRABICS TRACTATS D'ENTRE 6 I 12 ANYS.

Investigador principal: MsC Laura Asensio Jurado

Seu de la investigació:Universitat Politècnica de Catalunya i Hospital Universitari Mútua de Terrassa.

Ens dirigim a vostè per informar sobre un estudi en el qual se'l convida a vostè i al seu fill a participar. La nostra intenció és que rebi la informació correcta i suficient perquè pugui avaluar i jutjar si vol o no que el seu fill participi en aquest assaig. Llegeixi aquest full informatiu amb atenció i nosaltres li aclarirem els dubtes que li puguin sorgir.

DESCRIPCIÓ DE L'ESTUDI

Hem desenvolupat un estudi per a valorar l'efecte de visionar una pel·lícula en 3D en diferents variables de la funció visual. L'estudi consisteix en avaluar l'estereopsis, la desviació latent o manifesta, l'agudesa visual i la sensibilitat al contrast abans i després de la visualització de la pel·lícula El llibre de la selva. Aquesta, ha estat seleccionada amb criteris molt estrictes d'adequació al rang d'edat dels participants, i també per les seves característiques de disseny del 3D. La visualització d'aquesta pel·lícula no implica cap risc per al participant.

Aquest estudi contribuirà a un major coneixement de l'ambliopia i el seu tractament en la comunitat científica.

PROCEDIMENTS DE L'ESTUDI

Si el seu fill reuneix les condicions per a participar i accepten que hi participi, haurà de realitzar les següents proves i procediments:

1. Exploració optomètrica prèvia a la visualització de la pel·lícula.

2. Visualització de la pel·lícula ____________________ al cinema *Yelmo de Sant Cugat.*

3. Exploració optomètrica posterior a la visualització de la pel·lícula.

4. Qüestionari al finalitzar la visualització de la pel·lícula.

5. Exploració optomètrica als 3 mesos de la visualització de la pel·lícula.

PARTICIPACIÓ VOLUNTÀRIA

Ha de saber que la participació en aquest estudi és voluntària i que pot decidir no participar i retirar el consentiment en qualsevol moment, sense que per això s'alteri la relació amb el seu optometrista ni es produeixi cap perjudici en el seu tractament o seguiment.

CONFIDENCIALITAT

El tractament de les seves dades, la comunicació i la cessió de les dades de caràcter personal de tots els subjectes participants, s'ajustarà al que disposa el Reglament nº 2016 / 679 del Parlament Europeu i del Consell de 27 d'abril de 2016 relatiu a la protecció de les persones físiques pel que fa al tractament de dades personals i la lliure circulació de les dades, i a la Llei Orgànica 3/2018 del 5 de Desembre de protecció de dades personals i garantia dels drets digitals. Les dades recollides per a l'estudi estaran identificades mitjançant un codi i només els investigadors d'aquest estudi podran relacionar aquestes dades amb vostè i amb la seva història clínica.

Les dades recollides per aquest estudi seran registrades únicament mitjançant un codi de manera que no s'inclourà cap tipus d'informació que permeti identificar els participants. Només l'investigador de l'estudi i els seus col·laboradors amb el seu permís específic podran relacionar les seves dades amb la seva història clínica.

La seva identitat no estarà a l'abast de cap altra persona a excepció d'una urgència mèdica o requeriment legal. Podran tenir accés a la seva informació personal identificada, les autoritats sanitàries, el Comitè d'Ètica d'Investigació i personal autoritzat pel promotor de l'estudi, quan sigui necessari per a comprovar dades i procediments d'estudi, però sempre mantenint la confidencialitat d'acord amb la legislació vigent.

Només es cediran a tercers i a altres països les dades codificades, que en cap cas han de contenir informació que pugui identificar el pacient directament. En el cas que es produís aquesta cessió, seria per a la mateixa finalitat de l'estudi descrit i garantint la confidencialitat.

D'acord al que estableix la legislació esmentada, vostè pot exercir els drets d'accés, modificació, oposició i cancel·lació de dades. A més, ara també pot limitar el tractament de dades que siguin incorrectes, sol·licitar una còpia o que es traslladin a un tercer les dades que vostè ha facilitat per a l'estudi. Per a exercitar aquests drets, o si voleu saber més sobre confidencialitat, hauran de dirigir-se a l'investigador principal de l'estudi. Així mateix, té dret a dirigir-se a l'Agència de Protecció de Dades si no queda satisfet / a.

Les dades ja recollides no es poden eliminar encara que vostè abandoni l'estudi, per garantir la validesa de la investigació i complir amb els deures legals i els requisits d'autorització de medicaments. Però no es registraran noves dades si vostè decideix deixa de participar-hi.

Amb aquest document vostè ens dóna el seu consentiment perquè vostè i el seu fill participin en aquest estudi.

NOM I COGNOMS DEL PARTICIPANT

____

NOM I COGNOMS DELS PARES /TUTOR/A

____

DATE

--------/---------/---------

DATA FIRMA PARES / TUTOR/A

FIRMA INFORMANT

Laura Asensio Jurado Col 20.030

PRESSUPOST

Projecció especial al Cine Yelmo Premium Sant Cugat…………..… 844 euros

CURRÍCULUM VITAE DELS INVESTIGADORS

Laura Asensio Jurado

Estudiant de doctorat en enginyeria òptica a la Universitat d'Òptica i Optometria de Terrassa, en eL Centre de Desenvolupament de Sensors, Instruments i Sistemes (CD6) de la Universitat Politècnica de Catalunya. És llicenciada en Òptica i Optometria i Màster en Optometria i Ciències de la Visió per la Universitat Politècnica de Catalunya (UPC). Actualment també estudia psicologia a la Universitat Oberta de Catalunya (UOC). Desenvolupa la seva activitat clínica a l'Hospital Universitari Mútua de Terrassa, centrant la seva activitat professional principalment en optometria clínica pediàtrica i estrabisme. Les principals línies d'interès i recerca són el desenvolupament visual, principalment ambliopia i estrabisme.

Marc Argilés

Llicenciat en Òptica i Optometria, Màster en Optometria i Ciències de la Visió i Doctor en Enginyeria Òptica per la Universitat Politècnica de Catalunya (UPC), amb estada a la Hochschule für Technik FHNW, Institut für Optometrie (Olten, Suïssa).

Professor de la Facultat d'Òptica i Optometria de Terrassa (UPC), en les àrees d'optometria clínica i teràpia visual. Codirector de la segona i tercera edició del Màster en Optometria i Teràpia de la Visió en col·laboració amb la Fundació Politècnica de Catalunya i ACOTV.

Fellow de l'Acadèmia Europea d'Optometria i Òptica (EAOO) l'any 2018, forma part del grup de recerca del centre de desenvolupament de sensors, instrumentació i sistemes (CD6) de la UPC. Revisor científic de diverses publicacions.

Els seus interessos actuals se centren en la investigació sobre el tractament de les disfuncions binoculars i acomodatives mitjançant teràpia visual, i la caracterització dels moviments oculars i l'aplicació de videojocs d'acció a l'ambliopia.

Lluïsa Quevedo Junyent

Llicenciat en Psicologia i en Òptica i Optometria. És professora universitària del Grau d'Òptica i Optometria i del Màster en Optometria i Ciències de la Visió de la Universitat Politècnica de Catalunya (UPC).

Actualment coordina el programa de doctorat en Enginyeria Òptica de la UPC i va ser la directora tècnica del Centre de Visió del centre d'entrenament olímpic de Sant Cugat del Vallés (Barcelona).

Coautora de 25 articles en l'àmbit de l'optometria i la psicologia, així com de diverses contribucions a congressos nacionals i internacionals. Les principals línies d'investigació són la visió esportiva i e-sport, l'entrenament visual i les lents de contacte.ç

Clare Mestre

Clara Mestre es va llicenciar en Òptica i Optometria per la Universitat Politècnica de Catalunya l'any 2014 i es va doctorar en Enginyeria Òptica per la mateixa universitat l'any 2019. Després de la seva formació postdoctoral a la Indiana University School of Optometry, actualment és investigadora i professora a la School of Optics and Optometry de la Universitat Politècnica de Catalunya. Els seus interessos de recerca cobreixen la visió binocular, els moviments oculars i el desenvolupament visual.

Dennis M. Levi

Dennis Levi és professor a la Universitat de Califòrnia, Berkeley, amb nomenaments a l'Escola d'Optometria, The Graduate Group in Vision Science i l'Helen Wills Neuroscience Institute. La seva investigació se centra en com veiem la forma i la profunditat, i com aquestes es veuen afectades pel desenvolupament visual precoç anormal, especialment ambliopia i estrabisme. Ha rebut nombrosos premis, com ara el premi Glenn Fry, el premi Garland Clay i la medalla Prentice de l'American Academy of Optometry, i més recentment el premi Edgar D. Tillyer 2016 de la Optical Society of America, pel seu treball innovador en el tractament de l'ambliopia.

SUPORT AL PROJECTE I FINANÇAMENT

Aquest estudi forma part del projecte PID2020-112527RB-I00, finançat per MCIN/AEI/10.13039/501100011033
