# Supplementary material for: Can viewing a 3D movie improve visual function in children with a history of amblyopia and neurotypical children?: A pilot study
Source: PLoS One. 2024 Jun 25;19(6):e0305401. doi: 10.1371/journal.pone.0305401 (PMC11198783; doi:10.1371/journal.pone.0305401)
Supplement: S2 File — (PDF) [file pone.0305401.s005.pdf]

# VIEWING 3D MOVIE FOR THE RECOVERY VISUAL FUNCTION IN RESIDUAL AMBLYOPIC CHILDREN AND CHILDREN WITHOUT AMBLYOPIA

**RESEARCH PROTOCOL**

November 2019

## ABSTRACT

The aim of this study is to evaluate whether viewing an immersive 3D movie with large disparities in a cinema result in improved stereoscopic depth perception, ocular deviation, and visual acuity in 24 children aged 5 to 12 years with anisometropic and/or strabismic amblyopia previously treated with occlusion and neurotypical children without amblyopia. Measurements will be taken before viewing the movie, immediately after, and three months later. Our hypothesis is that viewing 3D films with large disparities will have an effect of improving stereopsis, visual acuity of the non-dominant eye and ocular deviation in previously treated amblyopic children aged 5 to 12 years.

GENERAL INFORMATION 6

RATIONALE AND BACKGROUND INFORMATION 7

REFERENCES 8

OBJECTIVES 9

GENERAL OBJECTIVE 9

SPECIFIC OBJECTIVES 9

STUDY DESIGN 9

METHODOLOGY 10

SAFETY CONSIDERATIONS 11

FOLLOW UP 12

DATA MANAGEMENT AND STATISTICAL ANALYSIS 12

QUALITY ASSURANCE 13

EXPECTED OUTCOMES OF THE STUDY 13

DISSEMINATION OF RESULTS AND PUBLICATION POLICY 14

DURATION OF THE PROJECT 14

PROJECT MANAGEMENT 14

ETHICS 14

INFORMED CONSENT FORMS 16

INFORMATION SHEET 16

INFORMED CONSENT 17

BUDGET 20

CURRICULUM VITAE OF THE INVESTIGATORS 20

SUPPORT FOR THE PROJECT AND FINANCING 21

## GENERAL INFORMATION

**Title: Viewing 3d movie for the recovery visual function in residual amblyopic children and children without ambliopia.**

**Name of investigators:** Laura Asensio-Jurado[1,2,3], Marc Argilés[1,2], Lluïsa Quevedo-Junyent[2], Clara Mestre[1], Dennis M. Levi[4]

**Addresses:**(1) Centre for Sensors, Instruments and Systems Development (CD6), Universitat Politècnica de Catalunya, Terrassa, Spain, (2) Departament d'Òptica i Optometria, Universitat Politècnica de Catalunya BarcelonaTech (UPC), Terrassa, Spain, (3) Hospital Universitari MutuaTerrassa, Terrassa, Spain, (4) Herbert Wertheim School of Optometry & Vision Science, University of California, Berkeley., CA, United States.

**Contact information:** Laura Asensio-Jurado, laura.asensio@upc.edu

**Telephone number:** +34636760450

## RATIONALE AND BACKGROUND INFORMATION

Amblyopia is a visual neurodevelopmental disorder associated, more frequently, with the presence of strabismus and anisometropia. It is clinically most important visual condition in childhood because, apart from refractive error, it is the most frequent cause of vision loss in children. It is a reversible condition that affects up to 2.9%[1] of the population, and that is the result of an abnormal visual experience during the most sensitive period of visual development. Although amblyopia is expressed in clinical practice as a reduction in visual acuity, it is also characterized by altered stereoscopy. Generally, amblyopia treatments focus on the recovery of visual acuity, and there are not many treatments that focus primarily on the recovery of altered stereopsis, which is so present and has a significant functional impact. New approaches have been developed that seek to restore binocular vision and focus on the rehabilitation of stereopsis in individuals with amblyopia and/or strabismus with encouraging results. These new approaches include perceptual learning[2-4], action video games[5-7], and 3D movie viewing[8]. The aim of this study reviewing, analyzing and, if applicable, updating the current treatment model for amblyopia. On the one hand, to evaluate the effectiveness of viewing a 3D movie in the improvement of stereoacuity, ocular deviation and visual acuity. On the other hand, to assess quantitatively the degree of satisfaction and acceptance of the participants and their families towards the intervention.

## REFERENCES

1 Fu, Z. *et al.* Global prevalence of amblyopia and disease burden projections through 2040: a systematic review and meta-analysis. *Br J Ophthalmol* 104, 1164-1170, doi:https://10.1136/bjophthalmol-2019-314759 (2020).

2 Li, R. W. *et al.* Sharpening coarse-to-fine stereo vision by perceptual learning: asymmetric transfer across the spatial frequency spectrum. *R Soc Open Sci* 3, 150523, doi:https://10.1098/rsos.150523 (2016).

3 Ding, J. & Levi, D. M. Recovery of stereopsis through perceptual learning in human adults with abnormal binocular vision. *Proc Natl Acad Sci U S A* 108, E733-741, doi:https://10.1073/pnas.1105183108 (2011).

4 Portela-Camino, J. A., Martín-González, S., Ruiz-Alcocer, J., Illarramendi-Mendicute, I. & Garrido-Mercado, R. A Random Dot Computer Video Game Improves Stereopsis. *Optom Vis Sci* 95, 523-535, doi:https://10.1097/opx.0000000000001222 (2018).

5 Levi, D. & Li, R. W. Playing 3-dimensional (3D), but not 2D video games can improve stereoacuity in neurotypical observers. *Journal of Vision* 19, 130a-130a, doi:https://10.1167/19.10.130a (2019).

6 Godinez, A., Martín-González, S., Ibarrondo, O. & Levi, D. M. Scaffolding depth cues and perceptual learning in VR to train stereovision: a proof of concept pilot study. *Sci Rep* 11, 10129, doi:10.1038/s41598-021-89064-z (2021).

7 Vedamurthy, I. *et al.* Recovering stereo vision by squashing virtual bugs in a virtual reality environment. *Philos Trans R Soc Lond B Biol Sci* 371, doi:https://10.1098/rstb.2015.0264 (2016).

8 Li, R. W. *et al.* Improving Adult Amblyopic Vision with Stereoscopic 3-Dimensional Video Games. *Ophthalmology* 125, 1660-1662, doi:https://10.1016/j.ophtha.2018.04.025 (2018).

## OBJECTIVES

### General Objective

- To study the effect on the visual function of viewing a 3D film in residual strabismic and/or anisometropic amblyopic patients aged between 5 and 12 years.

### Specific objectives

- Assess the effect of viewing a 3D film on stereopsis, visual acuity and eye deviation.
- Establish possible correlations between the different variables.
- To compare the effect of this intervention for subtypes of refractive and strabismic amblyopia.
- Provide data regarding the degree of satisfaction and feeling of parents and children in relation to the proposed intervention.

## STUDY DESIGN

A quasi-experimental study will be performed, without a control group, pre- and post-intervention in which subjects with refractive and / or strabismus residual amblyopia, that have been subjected to traditional treatment for amblyopia, will be selected. Variables of stereopsis, latent or manifest deviation and visual acuity will evaluate before viewing the movie, immediately after, and three months later. The sample will include subjects between 5 to 12 years old with a diagnosis of refractive and / or strabismic amblyopia. Children had undergone conventional treatment for amblyopia, occlusion or penalty treatment, before participation in the study.

Inclusion criteria will be: (1) prior diagnosis of strabismic and/or refractive amblyopia, (2) present a residual amblyopia defined as best-corrected visual acuity (BCVA) was ≥0.10

logMAR in one or both eyes and interocular difference in BCVA was ≥0.10, (3) amblyopia treatment completed at least 6 months before the intervention, (4) VA stability greater than or equal to 9 months, (5) absence of associated ocular pathologies, (6) not having seen a 3D movie before. In addition to the general criteria, the following inclusion criteria will be taken into consideration for the group with strabismus was: (1) present strabismus (2) angle of deviation equal or smaller than 35 PD; and for the group with anisometropic amblyopia was: (1) refractive difference of one diopter or more in the spherical or cylindrical component.

Neurotypical children from 5 to 12 years of age will be also recruited. The inclusion criteria for this group will be: (1) age between 5 and 12 years, (2) absence of amblyopia, (3) absence of other ocular pathologies, (4) not having seen a 3D movie before.

## METHODOLOGY

The present investigation is a pre-post-experimental prospective intervention study in which participants with refractive and/or strabismic residual amblyopia have previously followed a traditional treatment for amblyopia have been selected. Subjects who met the study's inclusion criteria will be informed and will sign consent for inclusion in the study. The sample includes participants between 5 and 12 years old evaluated by the optometry department of Hospital Universitari de Terrassa. The participants will be divided into three groups. The first group will include strabismic amblyopes, the second group will include refractive amblyopes, and the third group will include neurotypical children.

Before baseline assessment, all participants will undergo a comprehensive visual examination which will include: uncorrected visual acuity, retinoscopy and subjective refraction, best corrected visual acuity, near and far vision coverage test, near convergence point, valuation of extrinsic and intrinsic motility, evaluation of refraction under the effects of cycloplegic and macular and papillary examination using optical coherence tomography reviewed by an ophthalmologist.

The intervention will consist of viewing a 3D film. This will be selected for its 3D design, which is appropriate to the age of the participants and also because the film will be current and interesting for the participants in order to ensure their attention and motivation. The study will take place in the Yelmo de Sant Cugat cinemas and the film is yet to be finalized.

During viewing, participants will be their usual correction, previously reviewed and modified, if necessary, in the prior full visual examination. Patients with strabismus will not wear prisms. And, in addition, all participants will wear glasses with polarized lenses to watch the film in 3D.

To evaluate the effect of watching a 3D film on stereoacuity, monocular visual acuity and ocular deviation, the following test will be used. Stereopsis will be measured with the TNO test. The TNO test consists of 7 sheets for stereoscopic evaluation and should be used with red-green glasses. Monocular visual acuity of both eyes will be measured using the Snellen ETDRS E optotype at 3 m and the magnitude of the deviation will be measured with prism rods during the near (40 cm) and distance (6 m) cover test. All measuremens will be performed by texpert pediatric optometrists.

At the end of the intervention, the degree of satisfaction of the participants will be assessed using an ordinal questionnaire (from 1 to 5, with 1 being the least satisfaction and 5 the most satisfactory) based on the Questionnaire of satisfaction with medication treatment (TSQM) version 1.4.

## SAFETY CONSIDERATIONS

The data collected for the study will be identified by a code and only the researchers of this study will be able to link this data and the medical history. The data collected by this study will be recorded only by means of a code so that no information of any kind that allows the identification of the participants is included. Only the study investigator and

their collaborators with their specific permission will be able to link the data to the medical record.

The identity of participants will not be available to anyone else except for a medical emergency or legal requirement. Health authorities, the Research Ethics Committee and personnel authorized by the study sponsor may have access to your personally identified information, when necessary to check the data and procedures of the study, but always maintaining confidentiality in accordance with current legislation.

Only coded data will be transferred to third parties and to other countries, which must in no case contain information that can directly identify the patient. In the event that this transfer occurs, it will be for the same purpose as the study described and guaranteeing its confidentiality.

In accordance with the provisions of the aforementioned legislation, you can exercise the rights of access, modification, opposition and cancellation of the data.

## FOLLOW UP

Measurements will be taken before watching the film, immediately after and three months later.

## DATA MANAGEMENT AND STATISTICAL ANALYSIS

Qualitative variables will be described with percentages and quantitative variables with mean, median, standard deviation and maximum and minimum values. Clinical stereoacuity values in sec arc will be transformed into logarithmic units for analysis. Change in outcome variables from baseline to three months post-intervention will be analyzed using repeated measures ANOVA (RANOVA). Test sphericity will be considered using Mauchly's test and relevant corrections will be applied. SPSS version 27 for Windows will be used to perform the analysis.

## QUALITY ASSURANCE

This research follows and complies with GCP that guarantees patients their rights, safety and well-being in the study.

The processing of the data, the communication and transfer of the personal data of all participating subjects, will be carried out in accordance with the provisions of Regulation nº 2016/679 of the European Parliament and of the Council, of April 27, 2016, relating to the protection of natural persons with regard to the processing of personal data and the free circulation of data, and to Organic Law 3/2018, of December 5, on the protection of personal data and the guarantee of digital rights.

## EXPECTED OUTCOMES OF THE STUDY

In the pediatric optometric consultation, amblyopia is the most important condition to detect and treat to ensure correct visual and perceptual development, and thus promote correct learning and personal development. We have screening protocols in primary care to detect this condition early and start treatment. Passive occlusive therapy has been widely studied and its effectiveness in improving visual acuity has been demonstrated. Despite proven efficacy in children, results fail in more than 30% of cases, and those who respond to treatment often have residual amblyopia. In addition, occlusion has a high rate of non-compliance due to the fact that it is a long-term therapy and unattractive for children.

The introduction of new interventions in the treatment of amblyopia arises from the desire to offer an alternative to traditional treatment. On the one hand, they pursue the objective of improving treatment response time rates and recurrence rates. And on the other hand, it is intended to provide a treatment alternative more accepted by users by introducing recreational elements but at the same time incorporating other rehabilitators.

Even so, it is very attractive to propose that viewing 3D films with certain technical characteristics can translate into improvements in the psychophysical variables altered in amblyopia, and with greater acceptance among children

## DISSEMINATION OF RESULTS AND PUBLICATION POLICY

The results of the project will be disseminated in reference publications in journals and in international and national conferences. In addition, study participants and their families will be informed of the outcome of their different evaluations, as well as the final results of the study when they become available.

## DURATION OF THE PROJECT

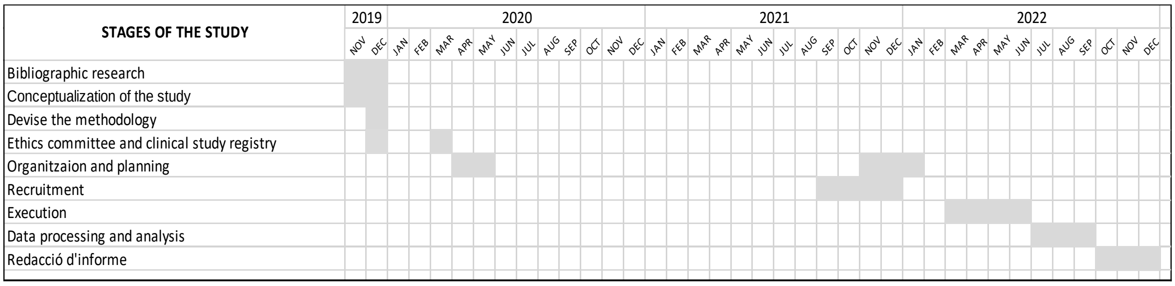


## PROJECT MANAGEMENT

Laura Asensio Jurado, Marc Argilés and Lluïsa Quevedo Junyent contributed to the conceptualization of the study, devised the methodology. They were part of the team in charge of the evaluations and data collection. They carried out data preservation and analysis. Subsequently and, together with Clara Mestres and Dennis M. Levi contributed to the data validation and the writing of the results.

## ETHICS

The study will be carried out in accordance with the Guidelines for Good Clinical Practice in Clinical Trial (2016) and the precepts of the Declaration of Helsinki (Brazil, 2013) on ethical principles for research involving human beings.

Patients and their parents or guardians will be informed of the nature of the study and must give their Informed Consent in writing, of which they will receive a signed copy. They will be clearly informed that they can leave the study at any time without this

affecting their follow-up or treatment. In the economic aspect, those involved in this study will not receive any type of compensation.

The confidentiality of patient data is guaranteed in accordance with Regulation nº 2016/679 of the European Parliament and of the Council of April 27, 2016 on the protection of personal data. The confidentiality of the data will be maintained by transferring it from the medical record to a coded database for analysis.

## INFORMED CONSENT FORMS

## <u>INFORMATION SHEET</u>

**Viewing a 3D movie can improve visual function in amblyopic and neurotypical children.**

The clinical study aims to evaluate the effectiveness in visual improvement after viewing a 3D movie, as well as the degree of satisfaction and interference in the child's daily routine. The treatment that must be carried out consists of watching a 3D movie. Before and after viewing the film, a visual evaluation will be carried out consisting of the measurement of visual acuity in far and near vision, stereopsis (3D vision) and the measurement of ocular deviation. In addition, at the end of the film there will be a small questionnaire to assess satisfaction in relation to the activity. After three months, a new visual evaluation will be offered to assess possible improvements

produced by this treatment. The film has been selected with very strict criteria of non-violence, being appropriate for the age of the participants.

For any information or clarification, you need, do not hesitate to contact me.

Laura Asensio Jurado

ambliopiatt@gmail.com

## INFORMED CONSENT

**Viewing a 3D movie can improve visual function in amblyopic and neurotypical children.**

Principal investigator: MsC Laura Asensio Jurado

Research site: Universitat Politècnica de Catalunya and Hospital Universitari Mutua de Terrassa.

We are contacting you to inform you about a study in which you and your child are invited to participate. It is our intention that you receive the correct and sufficient information so that you can evaluate and judge whether or not you want your child to participate in this trial. Read this information sheet carefully and we will clarify any doubts that may arise.

### DESCRIPTION OF THE STUDY

We developed a study to assess the effect of viewing a 3D film on different variables of the visual function. The study consists of evaluating stereopsis, ocular deviation and visual acuity before and after viewing the movie Sing 2. This has been selected with very strict criteria of suitability for the age range of the participants, and also for its 3D design characteristics. The viewing of this film does not involve any risk for the participant.

This study will contribute to greater knowledge of amblyopia and its treatment in the scientific community.

### STUDY PROCEDURES

If your child meets the conditions to participate and they agree to participate, they will have to perform the following tests and procedures:

1. Optometric examination prior to viewing the film.

2. Screening of the 3D movie __________________at the Yelmo cinema in Sant Cugat.

3. Optometric examination after viewing the movie.

4. Questionnaire at the end of viewing the movie.

5. Optometric examination 3 months after viewing the movie.

**VOLUNTARY PARTICIPATION**

You should know that participation in this study is voluntary and that you can decide not to participate and withdraw consent at any time, without this altering your relationship with your optometrist or causing any damage to your treatment or follow-up.

**CONFIDENTIALITY**

The processing of your data, the communication and transfer of the personal data of all participating subjects, will be in accordance with the provisions of Regulation nº 2016 / 679 of the European Parliament and of the Council of April 27, 2016 regarding to the protection of natural persons with regard to the processing of personal data and the free circulation of data, and to Organic Law 3/2018 of December 5 on the protection of personal data and the guarantee of digital rights. The data collected for the study will be identified by a code and only the researchers of this study will be able to relate this data to you and your medical history.

The data collected by this study will be registered only by means of a code so that no type of information that allows the identification of the participants will be included. Only the study investigator and their collaborators with their specific permission will be able to link your data with your medical history.

Your identity will not be available to any other person except for a medical emergency or legal requirement. The health authorities, the Research Ethics Committee and personnel authorized by the study sponsor may have access to your identified personal information, when necessary to check study data and procedures, but always maintaining confidentiality in accordance with current legislation.

Only coded data will be transferred to third parties and to other countries, which must in no case contain information that can directly identify the patient. In the event that this transfer occurs, it would be for the same purpose of the study described and guaranteeing confidentiality.

In accordance with the provisions of the aforementioned legislation, you can exercise the rights of access, modification, opposition and cancellation of data. In addition, you

can now also limit the processing of data that is incorrect, request a copy or transfer to a third party the data that you have provided for the study. To exercise these rights, or if you want to know more about confidentiality should be addressed to the principal investigator of the study. Likewise, you have the right to contact the Data Protection Agency if you are not satisfied.

Data already collected cannot be deleted even if you leave the study, to ensure the validity of the research and to comply with legal duties and medication authorization requirements. But no new data will be registered if you decide to stop participating.

With this document you give us your consent for you and your child to participate in this study.

NAME AND SURNAME OF THE PARTICIPANT

____________________________________________________________

NAME AND SURNAMES OF PARENTS OR GUARDIAN

____________________________________________________________

DATE

--------/---------/---------

SIGNATURE PARENTS / GUARDIAN/A.

INFORMING SIGNATURE

Laura Asensio Jurado Col 20.030

## BUDGET

Special screening at Cine Yelmo Premium Sant Cugat…..…………………..… 844 Euros

## CURRICULUM VITAE OF THE INVESTIGATORS

### Laura Asensio Jurado

PhD student in optical engineering at the University of Optics and Optometry of Terrassa, as part of the Centre for Sensors, Instruments and Systems Development (CD6) of the Universitat Politècnica de Catalunya. She has a degree in Optics and Optometry and a Master in Optometry and Vision Sciences from the Polytechnic University of Catalonia (UPC). She is also currently studying psychology at the Universitat Oberta de Catalunya (UOC). She carries out his clinical activity at the Hospital Universitari Mútua de Terrassa, focusing his professional activity mainly on pediatric clinical optometry and strabismus. The main lines of interest and research are visual development, mainly amblyopia and strabismus.

### Marc Argilés

Degree in Optics and Optometry, Master in Optometry and Vision Sciences and PhD in Optical Engineering from the Polytechnic University of Catalonia (UPC), with a stay at the Hochschule für Technik FHNW, Institut für Optometrie (Olten, Switzerland).

Associate Professor at the Faculty of Optics and Optometry of Terrassa (UPC), in the areas of clinical optometry and vision therapy. Co-director of the second and third edition of the Master in Optometry and Vision Therapy in collaboration with the Fundació Politècnica de Catalunya and ACOTV.

Fellow of the European Academy of Optometry and Optics (EAOO) in 2018, he is part of the research group at the center for development of sensors, instrumentation and systems (CD6) of the UPC. Scientific reviewer of several publications.

His current interests focus on research on the treatment of binocular and accommodative dysfunctions by vision therapy, and the characterization of eye movements and the application of action video games in amblyopia.

**Lluïsa Quevedo Junyent**

Degree in Psychology and in Optics and Optometry. She is a university professor in the Degree in Optics and Optometry and in the Master of Optometry and Vision Sciences at the Polytechnic University of Catalonia (UPC).

She currently coordinates the PhD program in Optical Engineering at the UPC and was the technical director of the Vision Center in the Olympic training center of Sant Cugat del Vallés (Barcelona).

Co-author of 25 JCR articles in the field of optometry and psychology, as well as several contributions to national and international conferences. The main lines of research are sports and e-sports vision, visual training and contact lenses.

**Clare Mestre**

Clara Mestre received her BS in Optics and Optometry from the Universitat Politècnica de Catalunya in 2014 and her PhD in Optical Engineering from the same university in 2019. After her postdoctoral training in the Indiana University School of Optometry, she is currently a researcher and lecturer in the School of Optics and Optometry of the Universitat Politècnica de Catalunya. Her research interests cover binocular vision, eye movements and visual development.

**Dennis M. Levi**

Dennis Levi is a Professor at the University of California, Berkeley, with appointments in the School of Optometry, The Graduate Group in Vision Science, and the Helen Wills Neuroscience Institute. His research focuses on how we see form and depth, and how these are impacted by abnormal early visual development, particularly amblyopia and strabismus. He has received numerous awards including the Glenn Fry Award, the Garland Clay Award and the Prentice Medal from the American Academy of Optometry, and most recently the 2016 Edgar D. Tillyer Award from the Optical Society of America, for his groundbreaking work on treating amblyopia.

## SUPPORT FOR THE PROJECT AND FINANCING

This study is part of the project PID2020-112527RB-I00, funded by MCIN/AEI/10.13039/501100011033
